# Sensing of minute airflow motions near walls using pappus-type nature-inspired sensors

**DOI:** 10.1371/journal.pone.0179253

**Published:** 2017-06-28

**Authors:** Christoph H. Bruecker, Vladimir Mikulich

**Affiliations:** 1 Department of Mechanical Engineering and Aeronautics, City, University of London, London, United Kingdom; 2 Institute of Mechanics and Fluid Dynamics, TU Bergakademie Freiberg, Freiburg, Germany; Centro de Investigacion Cientifica y de Educacion Superior de Ensenada Division de Fisica Aplicada, MEXICO

## Abstract

This work describes the development and use of pappus-like structures as sensitive sensors to detect minute air-flow motions. We made such sensors from pappi taken from nature-grown seed, whose filiform hairs’ length-scale is suitable for the study of large-scale turbulent convection flows. The stem with the pappus on top is fixated on an elastic membrane on the wall and tilts under wind-load proportional to the velocity magnitude in direction of the wind, similar as the biological sensory hairs found in spiders, however herein the sensory hair has multiple filiform protrusions at the tip. As the sensor response is proportional to the drag on the tip and a low mass ensures a larger bandwidth, lightweight pappus structures similar as those found in nature with documented large drag are useful to improve the response of artificial sensors. The pappus of a Dandelion represents such a structure which has evolved to maximize wind-driven dispersion, therefore it is used herein as the head of our sensor. Because of its multiple hairs arranged radially around the stem it generates uniform drag for all wind directions. While still being permeable to the flow, the hundreds of individual hairs on the tip of the sensor head maximize the drag and minimize influence of pressure gradients or shear-induced lift forces on the sensor response as they occur in non-permeable protrusions. In addition, the flow disturbance by the sensor itself is limited. The optical recording of the head-motion allows continuously remote-distance monitoring of the flow fluctuations in direction and magnitude. Application is shown for the measurement of a reference flow under isothermal conditions to detect the early occurrence of instabilities.

## Introduction

Sensing of low-speed air motions is of critical importance in nature for prey detection [1,2]. The term “minute” is understood as a small velocity magnitude in the order of several hundreds of μm s^-1^ which are signalling the presence of instabilities or disturbances in an otherwise calm situation or as addition to an otherwise quasi-steady flow situation. In nature, crickets are capable of sensing low-frequency flows by their sensory hairs down to a threshold of air velocities of about 100 μm s^-1^ [1,2] which can be seen as a lower bound of these minute air motions. In technical application, this is also important for monitoring minute amounts of air fluctuations as, e.g. for neonatal incubators to monitor infants in intensive care units [3]. Comfort of human ventilation is another field where it is necessary to ensure low air-speeds along the body for well-being, as e.g. for passengers in cars or aircrafts. Most of the measurement principles to measure air-flow speed use the method of hot wire [4] or optical flow detectors such as Laser Doppler Anemometry [5]. In the last decade, the method of a flexible fibre-type flow sensor has been developed [3,6] which uses the bending signal of the filament that is arranged normal to the flow as a measure proportional to the drag force acting along the filament which is proportional to the airflow velocity [3,6]. Attached to walls, those filaments sense the wall-shear stress when the assumption of a linear velocity profile is valid as a first approximation [6–8]. Especially for the comfort measurements, the direct sensing near the body is relevant since the flow near the hull of the body is already affected by the presence of the body itself, the location of the ventilation device as well as other internal objects in the room. Another field where the velocity detection near the wall is of importance is the investigation of near-wall turbulence. Herein, not only the mean flow but also the frequency content linked with instabilities of different time- and spatial scales is important to understand the physics of heat and momentum transfer in such flows. Instabilities in flows are typically evolving in a certain frequency range as waves with very low disturbance amplitude. Such instabilities in their early stage are difficult to be detected because of the low disturbance amplitude. This requires the sensors to be on one hand sensitive to small fluctuations in magnitude and direction of the air motions and on the other hand to have a constant frequency response over a broad range of frequencies. For the flexible single-hair wall-shear sensors reported above increasing sensitivity goes with the disadvantage of reduced bandwidth [6,7]. The pappus-type sensor as developed and used herein extends the single hair sensor with a tip which is built from multiples of filiform hairs as radial protrusions from the stem. The hundreds of hairs of the pappus increase the sensitivity of the sensor by increasing the drag at the tip. We make use of the availability of pappus structures of Dandelion seed in nature, which is a suited sensor size for our focus of research in large-scale convection flows. Until today the aerodynamics of pappus seeds has been studied with respect to their seed dispersal and drag has been measured in vertical drop experiments. Herein we use the drag in cross-wind direction for flow sensing while the foot of the stem of the pappus is fixated on a flexible membrane at the wall. It is not known if Dandelion use the pappus for sensing but all studies argue that the structure has evolved as a passive means to maximize dispersion with wind [9,10]. Therefore the use as the head of a flow sensor herein is new, while the objective to improve the sensor response is directly correlated with the aerodynamics related with maximization of dispersion. In nature, filiform hairs have evolved for instance in spiders for the sole purpose of sensing and can operate at fundamental limit of noise [1]. In principle, the structure of the Dandelion pappus can help to achieve such high resolution in artificial systems, especially if further scaled down. In addition, the multiple hairs on the stem may allow to operate the sensor also below the noise level as the multiple hairs filter the random noise and transfer only the coherent part of fluid motion.

The sensor is tested in a research facility for convection flows under controlled conditions of a reference flow and under exclusion of any external air-motions. This facility has practical restrictions which limits the number of tests. In general, a day-long period is necessary to achieve fully developed flow conditions once the temperature boundary conditions are set and all doors into the chamber are closed. Any variations of sensor location or installation require interim access into the inner of the chamber which causes disturbances and requires waiting hours to proceed with the tests. The use of an alternative sensor, e.g. electrical flow sensors, is not feasible due to wiring problems and non-approval when changing the walls. These circumstances apply partly also for incubators or environments with explosives gases. Thus, an optical sensing principle is the matter of choice herein which can be done by optical detection of the sensor tip motion from outside of the chamber through optical windows. As future studies with the sensors are planned for studying turbulent convection flows in the same facility the discussion in the following refers at some places back to these special requirements.

## Materials and methods

### Pappus sensor characteristics

**Fig 1:**
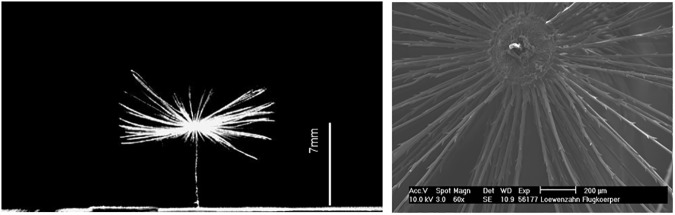
Pictures of the pappus sensor fixated with the stem at the wall. Pictures show the side view (left) with a scale bar and the top view with higher magnification (right).

We used the nature-grown pappus of Dandelion as a natural head of our sensor. The single-seeded fruit of Dandelion called achene is attached to a stem with a pappus of fine hairs at the tip, which ensure the maximization of wind-driven dispersal of the fruit. This pappus typically consists of hundreds of unbranched hairs of about diameter *d* = 20 – 30 × 10^−6^m that protrude outwards in radial direction [10–12] while their tip’s hull represents a segment of a sphere. The hairs arrangement and number density determines the permeability and influences the drag. For the Dandelion pappus used in our study the medium length of the hairs is 7mm and the total radial diameter *D* of the structure is about *D* = 14*mm*, see Fig 1. As discussed in [9] the typical Reynolds-number of the flow around the hairs in nature is of order of *Re*_*d*_ ≈ 1. So, drag forces are dominated by viscous friction rather than inertia.

**Fig 2:**
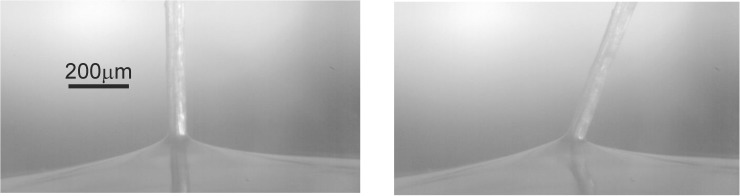
Foot fixation of the stem. The stem of the pappus sensor is fixated at the bottom wall in a thin silicone rubber membrane. Sensor at rest (left) and under strong wind load (right). Note that the stem keeps its straight shape under load.

After the fruit is cut off, the stem with the pappus is glued with rubber silicone (Polydimethylsiloxane, PDMS; Young’s modulus ≈1.5MPa) onto the wall of a squared flat metal sheet of 0.3mm thickness and a length of *L*_*ms*_ = 30*mm*. A silicone drop forms a flat membrane in which the stem foot is inset while being cured. A close-up view of the membrane with the stem is given in Fig 2. In its resting position the stem is perpendicular to the wall while under load the stem easily tilts to one side in the membrane and flexes back to its original position once being unloaded again. Thus the membrane mimics a torsional spring with which the stem is hold at the plate. As such, the senor represents the mechanical system of a wind-hair [1] with a torsional spring of spring constant k_M_ at the foot and with the stem plus the pappus at the tip as the drag-producing elements that force the stem to tilt under wind load. The mechanical model of such a structure describing the static and dynamic response is given in S1 Appendix, based upon earlier work on filament-type sensory structures as described in [1,2]. The impact of the viscoelastic response of the hierarchical pappus and the silicone rubber is illustrated by comparative step response experiments with the pappus sensor before and after cutting-off all hairs from the stalk as described in S1 Appendix.

Each sensor structure being hand-made this way represents an individual sensor with its own characteristics defined by the shape and arrangement of the pappus and the manual fixation process. The individual sensors are then handled as follows: firstly, they are subjected to an ex-situ mechanical characterization, secondly to an in-situ generated reference flow situation under isothermal wind-off conditions at different wall-jet speeds and directions. Finally, they are applied at the same location for sensing the flow in the same chamber at wind-on conditions for different settings of wall temperature boundary conditions.

For the purpose of the first tests, a systematic study of different nature-grown pappus structures was not practical. Each time one enters the research facility to change the sensor or the calibration velocity and direction, the internal flow is disturbed and it needs hours until the disturbances completely cease or the temperature boundary conditions are again under fully defined conditions. This disqualified up to now more tests of different hair structures and is left for future work when we can produce artificial pappus structures with defined and reproducible properties.

### Static calibration

The metallic base plate with the pappus sensor is tilted into the vertical plane such that the stem of the sensor is aligned with the horizontal. A static calibration of the spring stiffness k_M_ is done using weights hang on with a thin thread around the stem and measuring the tip deflection. The load position along the stem was varied and allowed us to determine the corresponding spring stiffness *k*_*M*_ of the fixation from the applied moment *M* and the measured deflection angle *θ* relative to the angle in equilibrium *θ*_*eq*_ without any external force. The torsional spring stiffness of the stem in the membrane is then calculated to *k* = *M*/*θ* as an average of all measurements, see S1 Appendix.

### Dynamic calibration

The dynamic response of the whole sensor structure is determined with a step response test, see S1 Appendix. Therefore, the metallic plate is oriented in horizontal plane and fixed with the floor. A thread is fixed with the head of the stem and the pappus sensor with the stem is then brought under tension in horizontal direction to a maximum tip excursion of 1.5mm. After abrupt instantaneous unloading, the flex back of the sensor tip is recorded with a high-speed camera at 5000fps in detail such that the path of the tip is fully captured in time. This signal is then passed into a Fast Fourier Transform (FFT) to determine the transfer function of the step-response. Because of the aerodynamic forces acting on the pappus, the reverse motion is aerodynamically damped in comparison to the situation of a single stem without any hairs, see S1 Appendix. The latter was done to isolate the effect of viscous relaxation of the membrane against the total relaxation of the sensor in full shape.

### Cross-wind drag

Compared to previous investigations, in the application herein the drag of the pappus is not measured against the vertical axis when it drops down but against a horizontal cross-wind direction. This is the load situation on the pappus sensor in our application as a sensor to detect horizontal air motion in the Ilmenau barrel, see below. To measure this cross-wind drag we used a thin paper of 150 micron thickness of square size (*L*_*ms*_ × *L*_*ms*_ = 30×30mm^2^, 120g/m^2^) the same size as the metal plate and fixed one pappus sensor at the centre of each side. The paper was then placed vertically in a 1.5 m long vertical stand with small slits on each side where the plate can glide down in vertical direction. The plate was then released from the top position and recorded while dropping. The mirror-symmetric pappus sensor arrangement on each side with their stems pointing in horizontal direction is then subjected to a cross-wind air motion relative to the axis of the stems. The terminal velocity *U*_0_ of the total body with mass *m*_*T*_ was measured and the drag force *F*_*D Pappus*_ was calculated from the force balance in direction of gravity

$$m_{T}\;g=F_{D\;Plate}+2\;F_{D\;Pappus}$$ (1)

The frictional drag force on the thin plate of planar area *A* is determined from the Blasius boundary layer theory [13] along a flat plate and the resulting drag coefficient *C*_*D*_

$$F_{D\;Plate}=\rho_{a}/2\;U_{0}^{2}{{\;A\;C}_{D}\;with\;C_{D}=1.328/\sqrt{{Re}_{L_{ms}}}}_{}$$ (2)

with air density *ρ*_*a*_ = 1,2 *kg*/*m*^3^. The Reynolds-number ${Re}_{L_{ms}}$ is defined as ${Re}_{L_{ms}}=U_{0}L_{ms}/\nu$ with the kinematic viscosity of air *ν* = 15 × 10^−6^ *m*^2^/*s* at room temperature. Measurements were repeated 10 times and average values were taken.

### Wall jet reference measurements

**Fig 3:**
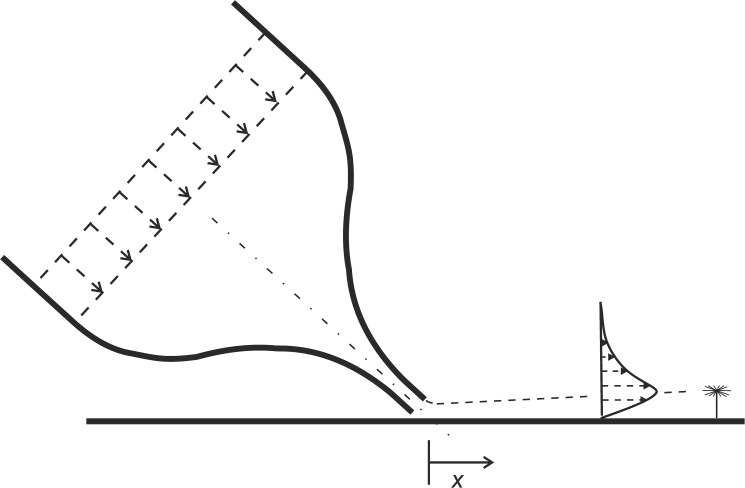
Sketch of the wall-jet apparatus. Air flow is generated with a planar nozzle flow that is tilted at an angle of *β* = 45° towards the bottom wall of the research facility and generates a wall-jet in direction of the sensor. The sensor is located at the centre of the bottom wall and the nozzle exit is at a distance of 20 slot heights *H* away. The 2D jet flow exiting the nozzle is pointing towards the sensor in *x*-direction.

An important aspect of the sensor is its usage to detect the near-wall flow in the barrel. Therefore, the sensor with its foot fixed at the wall is always subject to a velocity gradient which influences its response, depending on the velocity profile and the distribution of the hairs of the pappus. To incorporate this aspect in the reference tests in-situ, we studied the response of the sensor to a flow which is created by a wall-jet facility placed upstream of the sensor in the barrel. The wall-jet is a well-studied reference case for wall-bounded flows, the interested reader is referred to the following references [14–18]. It is also a typical feature of wall-bounded convection flows where bulk flow of the outer regions is directed towards the walls due to buoyancy forces. A similar flow situation can be generated with a jet exiting from a nozzle that is directed towards the wall. Further away from the nozzle exit along the wall the velocity profile in wall-normal direction approaches a self-similar solution and excellent agreement to the existing theoretical solutions has been reported for the laminar subcritical case [14–18]. A special case is the 2D wall jet which is generated with a rectangular nozzle of high aspect ratio between slot width and slot height. This kind of rectangular nozzle is used herein with a width of *W* = 120*mm* and a height of *H* = 5*mm*. All reference air-flow measurements in the research facility with the pappus sensor were carried out with the sensor placed at the centre of the bottom wall in the horizontal plane and subjected to the air-flow generated by the wall-jet facility as illustrated in Fig 3.

The origin of a Cartesian coordinate system fixed with the nozzle is defined at the bottom wall at the nozzle exit and the x-axis points in streamwise direction as given in Fig 3. The *y*-axis is normal to the wall and the *z*-axis points in the spanwise direction of the nozzle. Along the spanwise direction, the origin is located in the center of the nozzle. Flow is driven by a centrifugal fan at the entrance to the settling chamber, which is equipped with flow straightening devices screens [16]. After the settling chamber, the flow enters a smooth contraction of total ratio 20:1 and finally exits from the slot. Velocity profile measurements of the wall-jet flow field were obtained by Particle Image Velocimetry [19]. The velocity profile was measured at *x* = 20*H* downstream of the nozzle at the location where the sensor was positioned in the barrel. The velocity profiles were measured for different speeds of the fan.

### Measurement environment

All measurements with the wall-jet were done in the enclosed environment of the Ilmenau barrel. This environment is a large cylindrical enclosed chamber with a diameter of 7.0m and a maximum height of 6.3m [20]. The temperature of the bottom and top wall can be precisely controlled independently from each other. When set to isothermal conditions with all walls set to the same temperature, any remaining air-motion inside the barrel ceases after some period. If the walls are set to different temperatures one can achieve convection flows reaching large Rayleigh-numbers of maximum 10^12^ [20]. The top wall can be adjusted to different latitudes above the bottom one, in our experiments it was set to a level of 2m. The sensor is placed at the centre of the bottom wall and the wall-jet facility is placed with radial offset such that the sensor location is the distance *x* = 20*H* apart from the slot in the middle of the jet. Four different angular directions of the jet-flow towards the sensor were tested to investigate the sensor response against wind directions from north, east, south and west. For the calibration studies the barrel is set to isothermal conditions. There is no initial flow disturbance in the chamber before starting the fan of the wall-jet facility. Therefore, the wall-jet can be considered to operate in quiescent surroundings and the local air-motion at the sensor location is solely induced by the wall-jet facility for a certain period of time (of order several minutes). Later, the wall-jet facility is removed out of the barrel and flow studies with the pappus-sensor can be done under natural convection flow conditions set by the boundary conditions of the temperature controlled walls.

### Imaging the tip deflection

The sensor tip deflection in the horizontal plane is represented by the vector ***Q*** which gives the magnitude and direction of the shift under wind-load (wind-on) relative to its position at rest (wind-off situation) in the horizontal plane. The tip motion was recorded with a USB video camera (mvBlueVox, MatrixVision, 927x927 pixel^2^ at 10fps) from top outside of the isothermal chamber through a small glass window 2 meter apart from the sensor tip with a long-range microscope lens (K2/2 Infinity Photo-optical company). The recording format in the field of view was a squared cross-section of 7.8 × 7.8 mm^2^ centred with the axis of the sensor at wind-off. Illumination of the sensor is done with an expanded laser beam from another optical window in the top wall. Image processing using template cross-correlation methods with Gaussian subpixel analysis as in PIV [19] allowed detecting the shift between wind-off and wind-on situation with an uncertainty of about 0.05 pixel which is equivalent to a minimum resolved tip deflection of |***Q***| = 0.4 micron. In case of the mechanical response tests of the sensor structure we used a digital high-speed camera (Fastcam APX-RS, Photron, 512x512 pixel^2^ at 5000fps) to record the tip motion with high temporal resolution.

## Results

Each of the hand-made sensor structures represent an individual sensor with its own properties because of variation in nature-grown pappus hairs’ length and orientation in Dandelion seed and the fixation at the membrane. As described above a systematic variation study was not possible for practical reasons and artificial sensors with reproducible properties in the same size as the Dandelion seeds are not available up to now. Therefore in the following, the results are given mostly for a few selected sensors which were used throughout the whole measurement campaign. Experiments are done with sensors which have passed the mechanical load tests and the calibration in the reference flow in desired quality.

### Pappus sensor characteristics

The measurements of the bending load tests for the selected sensor gave a torsional spring stiffness of the stem in the membrane of about *k*_*M*_ = (305±10)×10^−9^ N m rad^-1^. As an example, a drag force of 1μN on the pappus leads to a deflection angle of about *θ* = 1.5° and a horizontal tip displacement of |***Q***| ≈ 210 micron at the tip of the stem 8mm apart from the wall. A typical drag force acting on the pappus in the cross-wind direction was measured to 2.4μN at a velocity of 0.5m/s. With the minimum resolved tip deflection of |***Q***| = 0.4 micron and the measured spring stiffness *k*_*M*_ the force detection threshold is about 1.9nN. If one assumes in first approximation a linear relationship between drag force and velocity for the low Reynolds-number flow around the hairs the detection threshold would be a velocity of about 400 μm s^-1^. This applies for the velocity acting on the pappus at an average wall-normal distance of 7mm apart from the wall. At flow speeds above 1.5m/s we observe the beginning of deformations of the hairs relative to each other and the deflection angle is larger than *θ* = 10°, which we set therefore as a upper bound for our mechanical tests. For calculation of the drag coefficient *C*_*D*_ the force was made dimensionless with the projection area of the pappus in cross-wind direction which is the area of an imaginary rectangular box formed by the pappus’ diameter *D* = 14mm and height of 4mm. This leads to a drag coefficient of *C*_*D*_ = 0.77 in cross-wind which is in the range documented by Casseau et al. (2015) [12] for vertical drop tests.

**Fig 4:**
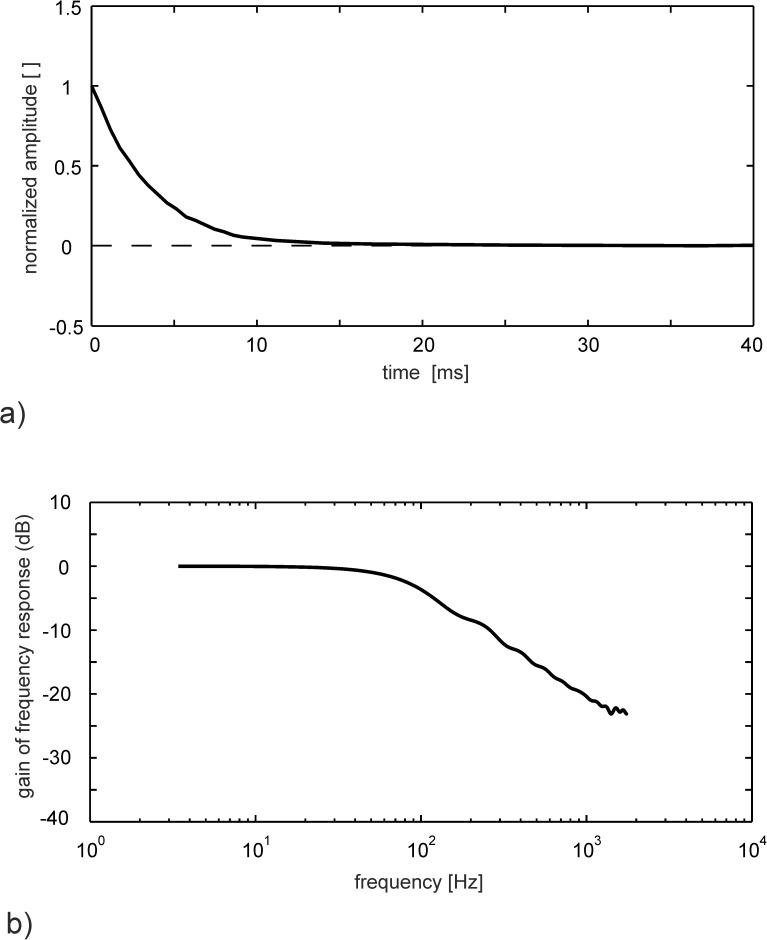
Sensor step response. a) tip motion back to rest position after abrupt unloading. b) sensor frequency response.

The dynamic response shown in Fig 4 exhibits a critically damped behaviour with a sensor response time of about *τ*_95_ = 0.01*s* and a constant response up to a frequency of about 100Hz, see S1 Appendix.

Beyond that, the plot shows a 20dB roll-off which is typical for an overdamped mechanical system [8]. The damping results mostly from the high aerodynamic resistance of the pappus in the relaxing motion. It is only marginally influenced by the internal damping of the membrane. This is illustrated by comparing the difference in step response behavior for the stalk with complete pappus and for solely the stalk after cutting-off all hairs, see also S1 Appendix.

### Wall-jet characteristics

**Fig 5:**
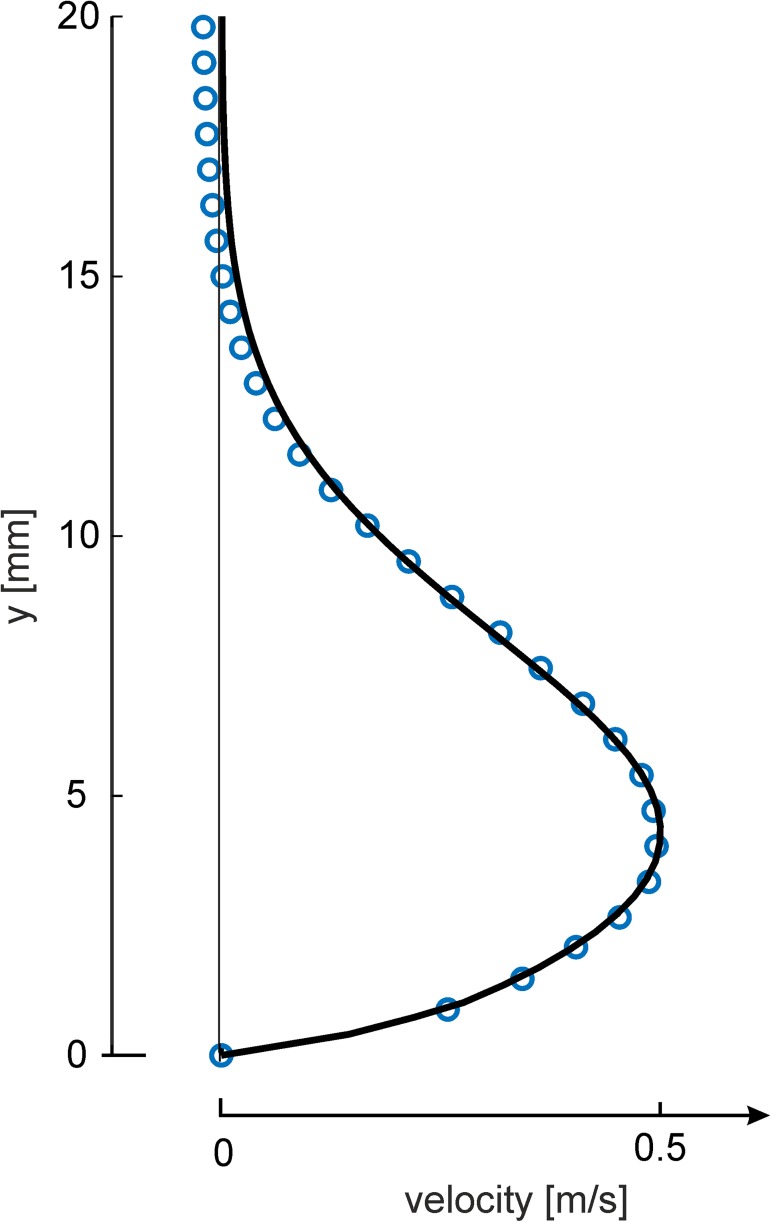
Velocity profile. Velocity profile of the wall-jet at *x =* 20*H* downstream of the nozzle (blue circles: measurements; solid line: fit with self-similar solution for a laminar wall jet, maximum velocity is *U*_0_)

The reference flow situation with the wall-jet generated in the isothermal chamber is used as an in-situ calibration to qualify the sensor for the intended studies of turbulent convection flows close to the walls of the large convection cell. Wall-bounded flows form a velocity profile along the wall-normal direction as observed in natural convection flow in the barrel near the walls, too. The wall-jet device is designed to generate a two-dimensional boundary layer flow directed towards the sensor with a similar velocity and scale at the location of the sensor as measured in earlier studies of convection flows in the barrel [20]. Focus of the tests of the sensor is the response against a quasi-steady flow situation at different flow direction towards the sensor. Such a quasi-steady flow at the sensor-location is achieved only for slot exit velocities between 0.3 and 0.6m/s. The lower bound is given by the fact that a certain limit of fan speed was required to generate a constant flow rate at the slot exit. On the other hand, above a slot exit velocity of 0.6m/s the jet flow starts to form vortices which pass the location of the sensor at *x* = 20*H* and generate fluctuations of large magnitude and rapid temporal changes which perturb the calibration process, see S1 Video. Therefore a typical setting for the exit velocity at the experiments is *U*_0_ = 0.5 m/s which is further discussed herein. The measured turbulence level of the outlet flow was less than 0.2% as obtained from PIV measurements close to the slot. These measurements showed also that after the start of the fan it takes about 6 seconds until the flow at the exit gets quasi-steady. The latency is due to the run-up of the fan and built-up of pressure in the wall-jet facility. The Reynolds-number defined with the slot height *H* is *Re*_*H*_ = 167. Bajura & Szewczyk (1970) experimentally obtain a very good agreement of the measured velocity profiles to the theoretical solution of the wall jet 18*H* downstream of the nozzle outlet for a jet-exit Reynolds number of *Re*_*H*_ = 377 [17]. As the flow Reynolds-number in our experiments is lower, the distance required to approach this solution also decreases [17]. The measured velocity profile is in good agreement with a fit with the self-similar Blasius jet solution [13] as shown in Fig 5.

The effect of wall inclination on the mean flow in a two-dimensional wall jet has been studied by Lai and Lu (1970) [18]. Their results indicate that in the range 0° ≤ *β* ≤ 45°, as the wall angle *β* increases, the centreline velocity decays faster, and the jet spreads faster, resulting in a shorter potential core and increase in jet volume entrainment.

**Fig 6:**
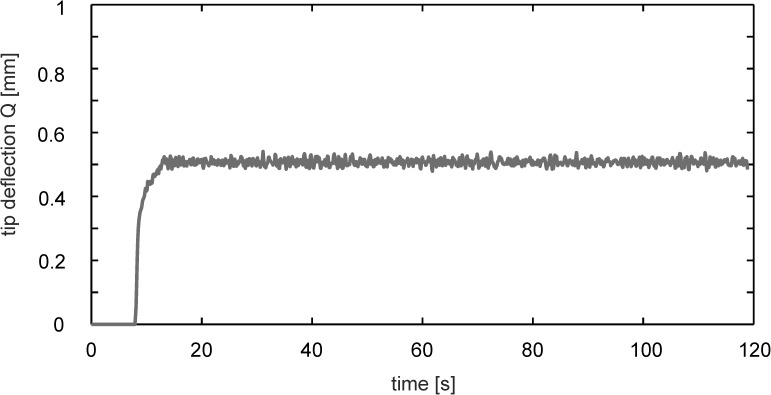
Time-trace of sensor tip motion in the wall-jet flow. Tip motion in streamwise direction after start of the fan of the wall-jet facility at *t* = 8*s* (*U*_0_ = 0.5 *ms*^−1^).

A typical record of the tip motion in streamwise direction as recorded with the camera from top is shown in Fig 6.

**Fig 7:**
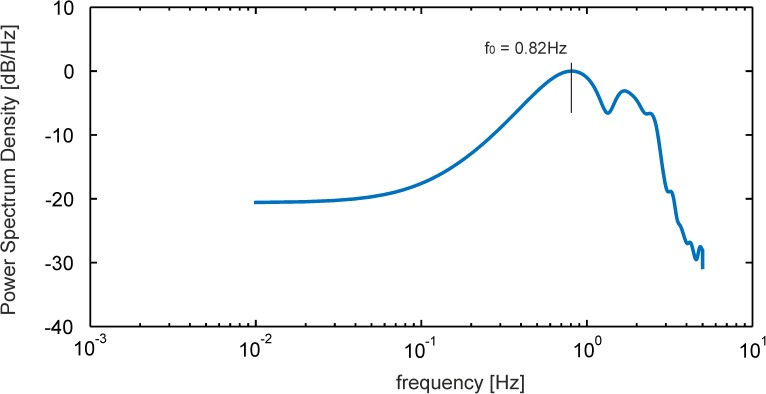
Power spectrum in the wall-jet flow. Power spectrum of the tip motion signal after start of the jet-flow in the quasi-steady state shown in Fig 6.

After the start of the fan at *t* = 0*s* one can see the above described latency of about 6 seconds until the flow velocity at the sensor position is approaching the constant mean. From *t* ≥ 14*s* on, the tip weakly fluctuates around a constant level of about 0.51 mm tip displacement until the end of the recording period of 120 seconds. The rms value of the tip motion after the initial transient is about 10micron which is 1.28 pixel size in the images of the recording camera. Given the time signal *Q*_*x*_(14*s*−120*s*) for the quasi-steady phase as shown in Fig 6, its power spectral density *PSD*(*f*) is defined as the Fourier Transform of the autocorrelation function of the signal. For reduction of noise in the estimated power spectra and to highlight the fundamental frequencies we used Welch’s method for spectrum estimation, in exchange for reducing the frequency resolution. The result is given in Fig 7.

The dominant peak is at a frequency of 0.82 Hz and the first harmonic is seen at 1.64Hz. The peak corresponds to the fundamental frequency of the primary instability of the jet as discussed below.

**Fig 8:**
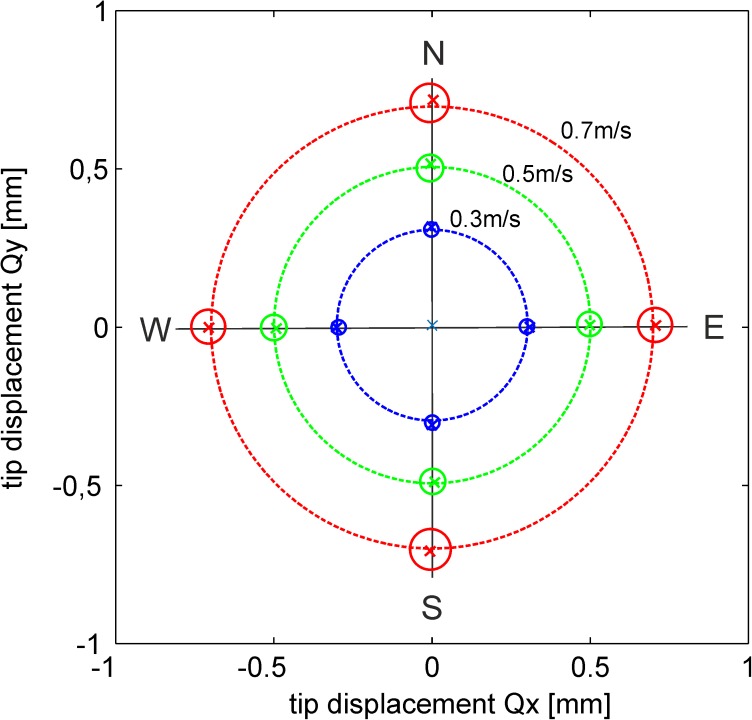
Polar plot of the directional tests. Directional response of the sensors for four different wind-directions generated with the wall-jet facility (north N, east E, south S, west W). The plot shows at the cross-symbols the mean response of the qualified sensor and the diameter of the small circles indicate the variation of all others being tested, partly failing to comply with our quality criterion. The larger dashed circles indicate the tested velocity of the jet flow. Note, that the tip displacement coordinates of Q are related to the x-y plane of the camera sensor parallel to the bottom wall. The coordinate system of the camera was rotated such that the direction to North is parallel to the positive y-direction in the plot.

The procedure was repeated for different exit velocities of the wall-jet in the range between 0.3 and 0.7m/s and the corresponding mean values of the tip displacement in streamwise direction were taken to calculate a linear regression through the origin with *U*_0_ [*m*/*s*] = 0.96 × 10^3^[1/*s*] * |***Q***| [*m*]. The resulting relation has an rms error of 0.01m/s in the investigated range of jet velocities. Based on the theoretically linear relation between drag and velocity for the low Reynolds-number flow around the tiny hairs it is assumed that this regression holds also for lower velocities which we however could not generate with the facility as a steady flow situation. The tests of the sensor at different flow directions of the wall-jet in north, east, south and west showed that the tip bending can vary sometimes up to 10micron at 0.5m/s jet velocity. This inequality is caused by a non-perfect bonding of the stem to the membrane which should ideally be such that the stem is exact perpendicular to the wall. Although maximum care was taken in this bonding process using the help of a three-axes micrometer traverse the result was not always perfect. Therefore the directional flow tests were used to select only those sensors for further measurements which show less than 4micron of tip bending difference, see Fig 8. This is equivalent to a velocity fluctuation of 0.01m/s, similar as the rms-error of the regression curve.

The observed increase of the variation with increase of jet velocity, especially beyond 0.6m/s, is partly due to the set-in of image blur when larger fluctuations occur at the higher jet velocities. This is because we are recoding in the calibrations with a frequency of 10Hz and the growing fluctuations in the jet flow at continuous illumination blur the image which deteriorate the peak detection algorithm. This can be circumvented with a high-intensity pulsed light source, which is now in use for the future measurements.

## Discussion

The calibration with the wall-jet flow is reproducible and tests with varying jet flow directions in the horizontal plane from north, east, south and west showed for the selected sensor structure only marginal differences of less than 4micron in tip bending for the different flow directions at maximum flow speeds of 0.7m/s. As the hairs of the pappus are directed radially outwards and their tips form the segment of a sphere, the drag onto the pappus is assumed to be indifferent to the angular direction of the flow in the horizontal plane. The nonetheless observed deviation is attributed to the bonding of the stem to the membrane which should ideally be the stem axis perpendicular to the wall. Only those sensors were finally used which passed the directional tests with sufficient small angular deviations.

The force on the pappus represents an average of the wind-induced drag along the individual hairs which see different speed and orientation against the local flow vector. Our measurements of the wall-jet flow therefore represent an average drag induced by the local velocity profile near the wall in a height of about 7mm at the tip the pappus. The integration of the velocity profile in wall-normal direction is over a length of ±2mm and in the wall-parallel plane along a radius of about 7mm. This integration effect limits the spatial resolution of the sensor in its current configuration with the Dandelion pappus, however the sensor size is still appropriate for the intended research of large-scale convection flows such as those in the Ilmenau barrel, where the smallest scales of fluctuations in studies at high Rayleigh-numbers up to 10^12^ were in the centimeter-scale, too [20]. We designed the wall-jet facility accordingly to generate a velocity and thickness of the boundary layer flow similar as observed in the barrel. Note that also much smaller pappus structures can be found in nature and can be used for our sensors, which however was not necessary for the purpose of the study.

The large number of small hairs of the pappus contributing to the total drag at the tip of the stem is the key to achieve the high sensitivity to minute air motions. The theoretical detection threshold in our configuration is a velocity of 400μm/s at the tip of the sensor which is a distance of 7mm away from the wall. Closer to the wall the velocity tends to decrease to zero which makes it increasingly difficult to sense the flow with smaller sensors. The detection threshold is given by the optical resolution of the imaging system which needs to resolve smallest differences in tip motion between wind-on and wind-off situation at a distance of 2m away from the sensory structure outside the barrel. Currently, this resolution is a tip-displacement of 0.4micron with the given optics. If the long-range microscope magnification is increased about a factor of 4 or alternatively a camera with a 16Mbyte pixel format would be used, the theoretical detection threshold would be 100μm/s. This is equivalent to the biological threshold reported in [1,2]. A combination of both, increased magnification and pixel format would even allow to measure with the sensor below the biological threshold.

The step response tests showed that the response time of the sensor is *τ_95_* = 0.01s. Further response tests were done with the pappus sensor before and after cutting-off all hairs from the stem. This comparison demonstrates that the viscoelastic relaxation in the membrane is not affecting the sensor response time for time-scales larger than the response time. With the sensor response time and detection threshold one can determine the Figure of Merit FOM as the inverse of the product of both. For the given optical conditions FOM = 0.25×10^−6^ m^-1^ which can easily be increased furthermore using higher resolution cameras and optics. As the dynamic sensor response is approximately constant up to frequencies of about 100Hz, it is qualified for investigations of turbulent flows containing coherent motions that vary in times-scales of 10ms and larger. Note, that the use of such pappus-type sensors for other applications and higher frequencies is possible, which requires the adaptation of the size and mechanical properties of the pappus and the membrane in a joint optimization process.

The sensitivity to minute air motions is demonstrated herein in Fig 7 as we could detect in the spectrum of the small tip fluctuations *Q*′ with an rms-value of 10micron a peak at a characteristic frequency of 0.82Hz which represents the most dominant mode of instabilities in the outer shear-layer of the two-dimensional wall-jet. This frequency agrees with the most amplified frequency of the Blasius shear layer predicted from theory. It is clear that under natural conditions, the wall jet operates in unforced mode and a broad band of frequencies it amplified. At the given Reynolds number of *Re*_*H*_ = 167, the outer mode (Blasius shear layer) is the only unstable mode of the Blasius wall jet [16]. The inner mode becomes unstable at a significantly higher Reynolds number of *Re*_*H*_ = 272 [16] which is higher than in the current experiment. The most amplified frequency of the Blasius shear layer predicted with inviscid stability theory by Monkewitz & Huerre (1982) is about *F* = 480 [21] where *F* is the reduced frequency defined as $F=10^{6}\;2\pi \;f_{0}\;\nu /U_{c}^{2}$ with the convective velocity *U*_*C*_. The latter is determined in our experiment at *Re*_*H*_ = 167 to *U*_*C*_ = 0.4 *ms*^−1^, measured from the velocity profile in Fig 5 at the location of maximum shear at *y* = 7 *mm*. The detected peak in the spectrum at *f*_0_ = 0.82 *s*^−1^ in Fig 7 is then at a reduced frequency of *F* = 483 which is close to the theoretical one documented above. This demonstrates that the pappus sensor is able to detect the weak disturbances of wave-type instabilities present in the quasi-steady wall-jet flow.

## Conclusions

Nature is used herein to build a pappus-type flow sensor which is applied to measure from remote distances optically minute air-flow motions near walls. The sensing principle is based on the measurement of transversal displacement of the tip of the pappus due to the drag force, which the air flow is inducing on it. Such a sensor structure mimics a single sensory hair which has additionally at the tip a large number of filiform protrusions, increasing the drag. The sensor presented herein has a pappus with 86 filiform hairs, each about 7mm long, taken from a nature-grown Dandelion fruit after mature, and used for near-wall airflow measurements in a large-scale research facility for investigations of natural and forced convection flows. For preparation of the sensor the single fruit called achene is cut-off from the stem and the stem with the pappus is then fixated at its foot with a flexible membrane onto the wall of a thin plate, which can be placed along the wall of interest. The joint of the stem with the membrane can be approximated as a circular isotropic bending spring and calibration allows to calculate the local velocity at the sensor tip; magnitude and direction is obtained from the tilting of the stem, which displaces the tip. This is recorded under wind-load relative to the situation at quiescent conditions over longer time-periods and provides the time-traces of the air-motion at the sensor location. The measurement uncertainty in magnitude of the local air-motion velocity of 0.01m/s is mainly determined by the calibration of the spring-constant and the integration effect of the drag along the wall-normal dimensions of the pappus for the applied wall-jet flow. The calibration provided a linear regression in the investigated range of jet velocities between 0.3 and 0.7m/s with velocity at the tip proportional to tip bending, which we assume can be extrapolated to lower velocities, giving the fact that—at the low Reynolds-number flow around the tiny cylindrical hairs–drag is linear proportional to velocity [13]. The relative error is definitely smaller as we could detect the early occurrence of the most dominant mode of the wave-type instability of the two-dimensional wall-jet from the frequency spectrum of the signal. This conclusion results from the fact that the recorded fluctuations in the signal were in magnitude lower than the absolute error. The theoretical detection threshold in the current imaging configuration is 400μm/s given by the limited resolution of the optical system, which can be further reduced even below the biological threshold using higher-resolution cameras and larger magnification optics.

The idea of improving the sensitivity of hair-like sensors by increasing the drag on the tip is obvious, however solutions with extra protrusions at the tip either may not practicable, or non-isotropic with the wind-direction or non-permeable. The latter especially induces pressure forces or shear-induced lift forces on the sensor, which interferes with the measurement of the drag force. In contrast, the pappus is still permeable to the flow while the hundreds of individual hairs on the tip of the sensor head maximize the drag for the costs of small additional weight. The latter causes a slight reduction in the bandwidth as the total mass of the sensor increases, but this leads to a clear gain in improved response and sensitivity. Further optimization requires the adaptation of the size and mechanical properties of the pappus and the membrane in a joint optimization process.

Note that the discussed results are obtained from the specific pappus taken from Dandelion seed with 7mm long hairs and for those sensors qualified in the reference flow of the two-dimensional wall-jet. The centimetre-size scale and the measured response time *τ*_95_ = 0.01*s* make those sensor suitable for the intended specific application of turbulence research in large-scale convection flow such as those studied in the Ilmenau barrel [20]. This is where the sensors were designed and tested for and where we could benefit from availability of nature-grown pappus structures. On the other hand, the access to the barrel gave us perfect conditions to test the sensors in the reference flow situation under exclusion of any external disturbances. A systematic study of a larger number of sensors in the reference flow was not practical up to now because of the specific circumstances to handle the sensors in this facility. Therefore, the given results are representative only for the specific size of the sensors and natural variability in orientation and number of hairs may lead to deviations.

The sensors which have passed the qualification process are currently used in-situ in the barrel for further studies of large-scale structures in turbulent convection flows under the conditions documented in [20]. Up to now, near-wall flow studies were difficult to achieve therein for long recordings periods under the given restrictions of the research facility. Our motivation to build such sensors is rooted in recent interest in the emergence of super-structures in such large-scale convection cells which might evolve in time-scales of hours and more. The long-time, remote monitoring of the near-wall flow with the developed sensors provides a practical way to detect such motions, as bandwidth and spatial as well as temporal resolution are sufficient.

Applications of such pappus sensors are in principle not limited to these conditions. Micro-fabrication technologies may help to design pappus sensors of various scales in a reproducible manner. This offers to vary systematically the structure of the sensors or to assemble arrays of sensors with the same properties. In addition, one might design the hair arrangement in the pappus tailored to specific measurement tasks such as the detection of certain events in the flow which is current work in our lab.

## Supporting information

S1 Video Recordings of the sensor with start of the reference flow (U_0_ = 0.66m/s). (WMV)

S1 Appendix Mathematical model, Determination of membrane spring stiffness and relaxation times of sensor and membrane. (DOCX)

S1 Datasets Sensor response to the wall-jet flow (U_0_ = 0.5m/s). txt file format (time in s, tip bending in pixel). (TXT)

S2 Datasets Details of the mechanical step response tests for the sensor with and without pappi at the tip. Excel file format. (XLSX)
