# Supplementary material for: Sensing of minute airflow motions near walls using pappus-type nature-inspired sensors
Source: PLoS One. 2017 Jun 28;12(6):e0179253. doi: 10.1371/journal.pone.0179253 (PMC5489159; doi:10.1371/journal.pone.0179253)
Supplement: S1 Appendix — (DOCX) [file pone.0179253.s002.docx]

**Appendix A1: Mechanical model of the pappus-sensor**

The mechanical model of the pappus follows closely the model developed for a single sensory hair given in [22,23]. The stem of the sensor is represented as a straight cylinder of length L standing vertically on the flat plate and rotating around its base in the viscoelastic membrane, see Fig A1-1.


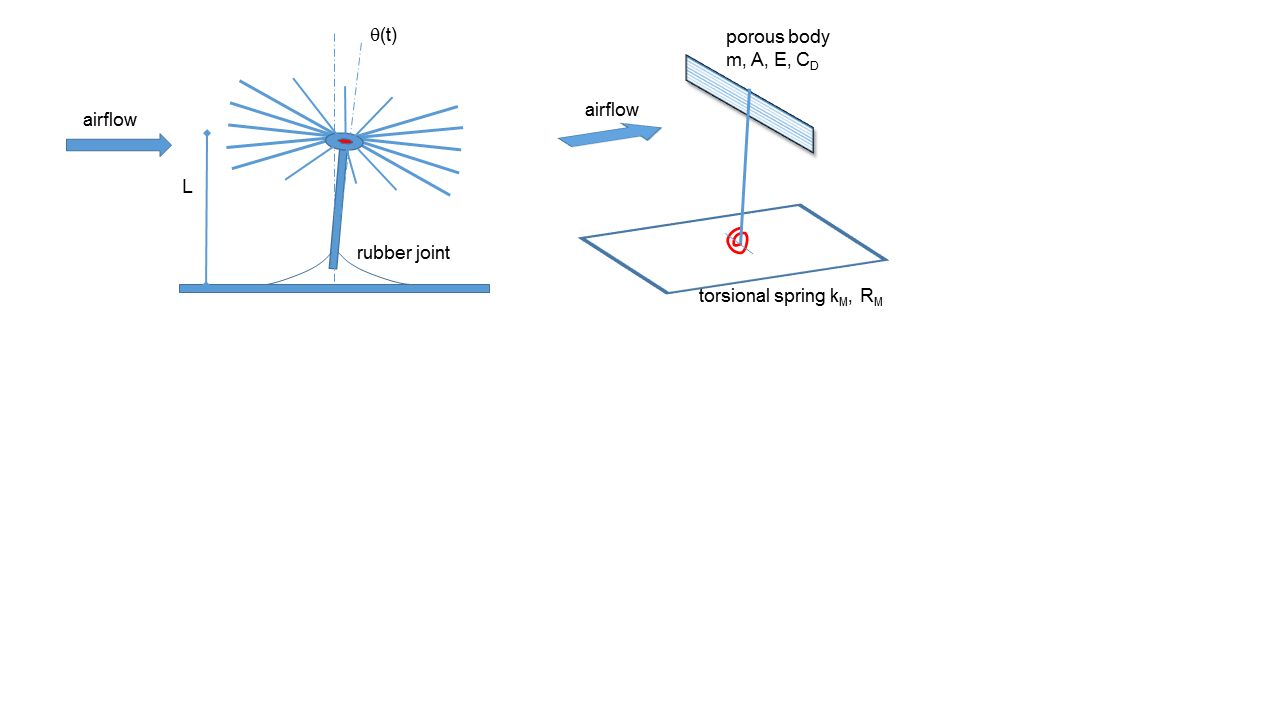


Fig. A1-1: Conceptual model of the pappus sensor and a simplified lumped element system with unidirectional orientation of the hairs perpendicular to the flow

The conservation of angular momentum for the motion of the stem with pappus is approximated as a forced, damped harmonic oscillator and is given by

(eq. A1-1)

$$I_{P}\ddot{\theta}+ R_{M}\dot{\theta}+ k_{M}\theta=M$$

where $\theta$ (rad), $\dot{\theta}$ (rad s^-1^) and$\ddot{\theta}$ (rad s^-2^) are the angular displacement, velocity and acceleration of the pappus about its pivot point. The moment of the inertia of the pappus is $I_{P}$ (N m s^2^ rad^-1^); the joint within the membrane is modelled as a linear viscoelastic torsional element that resists pappus deflection with a torque $k_{M}\cdot\theta$ and also experiences a frictional torque $R_{M}$, reflecting the rotational energy dissipated at the joint. This is illustrated in the simplified lumped element model shown in Fig. A1-1 with unidirectional horizontal orientation of the hairs. $M$ (N m) is the total torque acting on the pappus and is composed from different forces. For a single vertical hair standing out from the joint the torque $M$ is given as follows

$M= \int_{0}^{L} \left( f_{AM}+f_{D} \right) ydy$

(eq. A1-2)

where $f_{AM}, f_{D}$ are forces per unit length acting on the hair [22,23]. The first term is known as the added mass- or hydrodynamic mass force due to the acceleration of the near surface flow in the vicinity of the accelerating body, and the second term is the drag force.

In principle, those terms need to be integrated from the joint along the stalk and along all pappus hairs to build the total torque around the pivot. For further analysis of the relative importance of the terms, we simplify the situation of the flow around the pappi as flow around a porous body of volume $V$ and mass $m_{P}$ with a given frontal area $A$ and porosity $E$, see figure A1-1. Then the torques on the right hand side can be simplified in first approximation as resulting from point forces acting on the centre of the body:

(eq. A1-3)

$$M \approx\left. \left( \bar{F}_{AM}+ \bar{F}_{D} \right) \right|_{point} L$$

The added mass force is given by the acceleration of the volume of air of density $\rho_{a}$ surrounding the hairs. Usually this volume of air is assumed to be the same as that of the body:

(eq. A1-4)

$$\bar{F}_{AM}=\left( 1-E \right){V\rho_{a}\dot{u}_{r}= m}_{a} \dot{u}_{r}= m_{a}\left( \dot{u}_{a}-L\ddot{\theta} \right)$$

The drag force is the sum of the drag acting on all individual hairs, in total a number of $N$ hairs. For further analysis we assume that the individual hairs are identical slender cylinders with diameter $d$ and length $l$ which are normal to the flow in horizontal alignment. The cylinders see a velocity profile $u_{a}\left( y \right)=u_{a}\left( L \right)\cdot y/L$ which is in first approximation linear to the wall distance with $U_{0}$ being the velocity at the tip of the stalk. In addition, the centre of force is chosen as the centre of the body at the tip of the stalk. Both, the length of the cylinders $l$ as well as the length of the stalk $L$ are of the same order. The drag force acting on the stem is in first approximation neglected relative to the drag acting on the pappus. For low Reynolds-number flow around the pappus hairs the drag on each hair can be estimated by the classical Oseen relation of the drag on a cylinder with the axis perpendicular to the flow, compare also [22,23]:

(eq. A1-5)

$$F=\pi\frac{\mu}{2}ul$$

The sum over all $N$ pappus hairs results into a drag force

(eq. A1-6)

$$\bar{F}_{D}= \frac{1}{L}\sum_{i=1}^{N} \pi\frac{\mu}{2}\left( u_{a}(y_{i})-y_{i}\dot{\theta} \right)l_{i}y_{i}\approx N\pi\frac{\mu}{2}\left( u_{a}(L)-L\dot{\theta} \right)L$$

The lumped element model transforms equation A1-1 into

(eq. A1-7)

$$\left( I_{P}+I_{a} \right)\ddot{\theta}+ \left( R_{M}+R_{a} \right)\dot{\theta}+ k_{M}\theta=M_{F}$$

with the moment of inertia of the added mass

(eq. A1-8)

$I_{a}=m_{a}L^{2}$,

the frictional resistance of the motion of the pappus hairs

(eq. A1-9)

$R_{a}=N\pi\frac{\mu}{2}L^{3}$,

and the forcing torque

(eq. A1-10)

$M_{F}=m_{a} \dot{u}_{a}L+ N\pi\frac{\mu}{2}u_{a}L^{2}$ .

A non-dimensional form of A1-7 is obtained by scaling the variables $t, u$ and the displacement $s$ at the tip of the sensor for small angles $\theta$as follows:

(eq. A1-11)

$$t´=\frac{t}{T_{f}} , u´=\frac{u}{U_{0}} , s´=\frac{\theta L}{d}$$

where $T_{f}$ is the characteristic time-scale of an instability wave or the turn-over time of a vortex structure in the flow and $U_{0}$ is the characteristic jet velocity. The transformation of A1-7 with the dimensionless variables after dividing through $I_{P}$ and multiplying with $T_{f}^{2}$ reads:

$$\left( 1+\frac{\rho_{a}}{\rho_{P}} \right){s'}_{t´t´}+\left( \frac{T_{f}}{T_{MR}}+\frac{T_{f}}{T_{FR}} \right){s'}_{t´}+\left( \frac{T_{f}}{T_{O}} \right)^{2}s'=\frac{\rho_{a}}{\rho_{P}}\left( \frac{U_{0}T_{f}}{d} \right){u'}_{t´}+\left( \frac{T_{f}}{T_{FR}} \right)\left( \frac{U_{0}T_{f}}{d} \right)u'$$

(eq. A1-12)

with the pappus damping time constant $T_{FR}$due to aerodynamic resistance

(eq. A1-13)

$$T_{FR}= \frac{I_{P}}{R_{a}}= \frac{I_{p}}{N\pi\frac{\mu}{2}L^{3}} ,$$

the internal damping time constant $T_{MR}$ of the membrane

(eq. A1-14)

$T_{MR}= \frac{I_{P}}{R_{M}}$,,

and the inertia time scale $T_{0}$ characterising the period of oscillation in undamped motion

(eq. A1-15)

$$T_{O}=\sqrt{\frac{I_{P}}{k_{M}}} .$$

The resulting non-dimensional group contains the following five parameter:

$\Gamma= \frac{\rho_{a}}{\rho_{P}} \Lambda= \frac{T_{f}}{T_{O}} \Psi=\frac{T_{f}}{T_{MR}} \Pi= \frac{T_{f}}{T_{FR}} \Phi=\frac{U_{0}T_{f}}{d}$

(eq. A1-16)

Note that these parameter are the inverse of the ones given in [22] because of different normalization of equation A1-7. It is done here with the reference force of the hair inertia. The above given time-scales can easily be measured from step response tests of the sensor.

In eq. A1-16 Γ is the density ration, Λ is the ratio of flow time scale to the pappus inertial time scale and Ψ is the flow time scale relative to the relaxation time constant of the joint. A similar ratio Π is the flow time scale relative to the aerodynamic damping time constant. Note that this ratio appears on the left side of the equation as a damping factor and on the right-hand side of the equation as a factor related to the viscous driving force. Finally, Φ is the ratio of the time scale of flow relative to the convective flow time scale $d/{U_{0}}$ over the hairs. Thus the non-dimensional form of eq. A1-7 reads

(eq. A1-17)

$$\left( 1+\Gamma\right)\ddot{s}+\left( \Psi+\Pi\right)\dot{s}+\Lambda^{2}s=\Phi\left( \Gamma\dot{u}+ \Pi u \right)$$

An order of magnitude estimation follows herein. Typical velocities of the air flow in the boundary layer are of order $U_{0}\mathcal{=O}\left( {10}^{-1} \right) m s^{-1}$and characteristic turn-over times of the smallest vortex structures in the flow are $T_{f}\mathcal{=O}\left( {10}^{-1} \right) s$ which also represent the highest frequencies measured so far in the turbulent convection flow in the barrel [20]. A characteristic diameter of the pappus hairs is of order of$d= \mathcal{O}\left( {10}^{-5} \right) m$so that$\Phi\mathcal{= O}\left( {10}^{3} \right)$. The density ratio of air to the material of the hairs is about $\Gamma\mathcal{= O}\left( {10}^{-3} \right)$. The membrane tests in Appendix A4 gave a membrane relaxation time of $\tau_{1}= 3.3\times{10}^{-3}=2 T_{MR}$ which yields $\Psi\mathcal{= O}\left( {10}^{2} \right)$. The aerodynamic relaxation time of the sensor measured in the step response experiments is $\tau_{S}=3.3\times{10}^{-3}s$ which results in $\Pi\mathcal{= O}\left( {10}^{2} \right)$. Finally, the measured period of oscillation is $3.5\times{10}^{-3}s=2\pi T_{0}$ which leads to $\Lambda\mathcal{= O}\left( {10}^{2} \right)$.

Dropping the terms of lowest order $\mathcal{O}\left( {10}^{0} \right)$in eq. A1-17 leaves only terms of order $\mathcal{O}\left( {10}^{2} \right)$ and higher which shows that the motion behaviour of the pappus-sensor is represented by a first order damped mechanical system with the forcing term on the right hand side being proportional to the incident flow velocity:

(eq. A1-18)

$$\left( \Psi+\Pi\right)\dot{s}+\Lambda^{2}s=\Phi\Pi u$$

Thus the equation of motion A1-7 can be approximated as

(eq. A1-19)

$$\frac{\left( R_{M}+R_{a} \right)}{k_{M}}\dot{\theta}+\theta=\frac{R_{a}}{k_{M}L}U_{0}$$

which is of the form of

(eq. A1-20)

$\tau_{S}\dot{\theta(t)}+\theta\left( t \right)=CU_{0}\left( t \right) ,$ $\tau_{S}=(R_{a}+R_{M} )/k_{M}$

Here the time $\tau_{S}$ is the sensor relaxation time. Another time-scale is the response time $\tau_{95}$ which is defined as the time required to reach 95% of the final equilibrium position and can be calculated from $\tau_{S}$.

For the given pappus-sensor structure within the membrane joint, the order or magnitude estimation shows that internal viscoelastic and viscous drag forces dominate pappus inertia and added mass forces. Therefore the sensor tip displacement is in phase and proportional to the incident flow velocity at the tip of the stalk as long as the sensor relaxation time $\tau_{S}$ is small compared to the characteristic time scale of the flow $T_{f}$, a conclusion which has been drawn also for single sensory hairs of small size [21]_._

**Appendix A2: Determination of membrane stiffness** $\boldsymbol{k}_{\boldsymbol{M}}$

For determination of the membrane’s torsional spring constant $k_{M}$ the equation of motion is used in static form where all time-dependent terms cancel to zero and the driving torque $M_{ext}$ is solely given by the applied weight and the lever relative to the joint. Therefore equation A1-1 reduces to

(eq. A2-1)

$$k_{M}\theta=M_{ext}$$

The measurements were done with a number $n$ of tests using the same weight of mass $m$ at different levers $l_{i}$ relative to the joint and the angle $\theta_{i}$ was measured relative to the angle $\theta_{eq}$ in equilibrium position. The average was taken to determine the spring constant:

(eq. A2-2)

$$k_{M}=\frac{1}{n}\sum_{i=1}^{n} mgl_{i}/\left( \theta_{i}-\theta_{eq} \right)$$

**Appendix A3: Determination of sensor relaxation time** $\boldsymbol{\tau}_{\boldsymbol{S}}$

The sensor time constant is obtained by a step response test. Therefore the sensor is tilted to one side at angle θ_0_ and then released to relax back to its equilibrium position $\theta_{eq}$. This represents a step response to a heavyside step of $M_{ext}\left( t<0 \right)=M_{0}$ and $M_{ext}\left( t\geq0 \right)=0$. Fig. A3-1 shows the measured response curve. It clearly demonstrates the transient character represented by the first order approximation in eq. A1-20. The response is described by the homogeneous solution of eq. A1-20:

(eq. A3-1)

$$(\theta\left( t \right)-\theta_{eq})/{(\theta}_{0}-\theta_{eq})= e^{-t/ \tau_{S}}$$

For small angles the angular motion is proportional to the transversal tip motion $s(t)$ which is tracked from the images and measured relative to the tip coordinate in equilibrium position, normalized with the maximum excursion. The best fit to a function of type $s\left( t \right)=e^{-t/\tau}$ provides a relaxation time of the sensor of $\tau_{S}=3.3\times{10}^{-3}s$. The settling time or response time $\tau_{95}$ is defined as the time required to reach 95% of the distance to the final equilibrium position and is calculated to $\tau_{95}=10\times{10}^{-3}s$.

Fig A3-1: Step response of the sensor in the membrane after release (symbols: measurements, solid blue curve: curve fit $s(t)$). Because of manual tracking of the tip in the high-speed recordings the measurement data in the signal are not equidistant in time.

**Appendix A4: Determination of membrane relaxation time** $\boldsymbol{\tau}_{\boldsymbol{1}}$

The viscoelastic relaxation of the membrane is obtained by a step response test as in A3. However, now the hairs on the stalk were cut-off from the stalk leaving only the stalk in the membrane (note that this was done after the airflow measurements were finished).

Fig A4-1: Step response of the stalk in the membrane after release (symbols: measurements, solid blue curve: curve fit $s(t)$, solid red curve: curve fit $s_{2}(t)$. Because of manual tracking of the tip in the high-speed recordings the measurement data in the signal are not equidistant in time.

The obvious oscillatory motion in comparison to the overdamped behaviour of the sensor shown in Fig 4 is the result of the largely reduced aerodynamic damping since the hairs are completely removed from the stalk. The mechanical systems now represents a second order damped oscillator. The theoretical solution to the step function is

(eq. A4-1)

$$(\theta\left( t \right)-\theta_{eq})/{(\theta}_{0}-\theta_{eq})= e^{-t/\tau} cos\left( 2\pi t/T \right)$$

For small angles the angular motion is proportional to the transversal tip motion which is tracked from the images. The tip displacement $s(t)$ is measured relative to the tip coordinate in equilibrium position and is normalized with the maximum excursion of the oscillatory motion.

Overlaid on the damped oscillatory motion is the viscoelastic transient relaxation of the rubber membrane which is shown by the red line in Fig A4-1. The overall behaviour in Fig. A4-1 is approximated as the sum of a first order transient relaxation motion $s_{1}(t)$ plus the solution of the second order damped oscillation $s_{2}(t)$ as follows:

(eq. A4-2)

$$s\left( t \right)=s_{1}\left( t \right)+s_{2}\left( t \right)= {\hat{s_{1}}e}^{-t/\tau_{1}}+ {\hat{s_{2}}e}^{-t/\tau_{2}}\cos\left( 2\pi t/T \right)$$

The best fit to the measured profile is obtained for $\tau_{1}=3.3\times{10}^{-3}s (\hat{s}_{1}=0.25)$, $\tau_{2}=3.7\times{10}^{-3}s (\hat{s}_{2}=1)$ and $T=3.5\times{10}^{-3}s$. Note that the relaxation time constant of the membrane is equal to the measured relaxation time of the pappus-sensor, thus the viscous relaxation of the membrane has no further influence on the sensor response as long as the flow fluctuations occur at time scales larger than the response time of the sensor.
